# Awareness, practices and perceptions of community pharmacists towards antimicrobial resistance and antimicrobial stewardship in Libya: a cross-sectional study

**DOI:** 10.1186/s40545-023-00555-y

**Published:** 2023-03-21

**Authors:** Hiba A. Al-Shami, Usman Abubakar, Maryam S. E. Hussein, Hanin F. A. Hussin, Sondos A. Al-Shami

**Affiliations:** 1 Anaesthesia and Emergency Medicine Department, College of Medical Technology, Benghazi, 141 Benghazi, Libya; 2 Department of Clinical Pharmacy and Practice, College of Pharmacy, QU Health, Qatar University, Doha, Qatar; 3 Department of Pharmacology and Toxicology, Faculty of Pharmacy, University of Benghazi, 141 Benghazi, Libya; 4 Faculty of Pharmacy, Libyan International Medical University, 141 Benghazi, Libya

**Keywords:** Antimicrobial resistance, Antimicrobial stewardship, Practices, Perceptions, Community pharmacists, Libya

## Abstract

**Background:**

Community pharmacists play a vital role in promoting appropriate use of antibiotics in the community. This study evaluated the practices and perceptions of community pharmacists towards antibiotic use, antibiotic resistance, and antimicrobial stewardship in Libya.

**Methods:**

A cross-sectional study was conducted among community pharmacists in Libya using a 47-item validated, pre-tested online questionnaire. Data was collected from December 2021 to February 2022 and was analysed using descriptive and inferential analyses.

**Results:**

Of the 114 questionnaires included in the analysis, 54.4% were females, 78.1% had < 10 year working experience (78.1%), and 81.6% had a Bachelor of Pharmacy. Most (78.1%) strongly agreed/agreed that community pharmacists have an important role to play to reduce antibiotic resistance. Overall, the participants had a moderate perception towards antimicrobial resistance (median score: 21.5; IQR [16–28] out of 35.0). More than 40% supply antibiotics when patients requested them specifically, because if they do not supply them, patients will just go to another pharmacy. About 47% strongly agreed/agreed that if a patient cannot afford a full course of antibiotics all in one go, they will give them a smaller amount that they are able to afford at that time, even when a longer duration of treatment is required. Most (66.7%) had no previous involvement in antibiotics awareness campaign due to ‘I have never heard about the campaign’ (48.7%) and ‘I do not have enough time to participate’ (22.4%). Overall, the perception towards antimicrobial stewardship was good (20 [13–25] out of 25.0). Raising awareness of rational antibiotic use, including antimicrobial resistance, among pharmacy students (83.4%) and patient education by pharmacists in community pharmacies at the time medicines are supplied to patients (81.6%) were the most common strategies to improve rational use of antibiotics in community pharmacy.

**Conclusions:**

Community pharmacists in Libya recognise their role in reducing antimicrobial resistance. They had a moderate perception towards antimicrobial resistance and a good perception towards antimicrobial stewardship. However, inappropriate antibiotic practices were common. The most common strategies to improve rational use of antibiotics in community pharmacy were raising awareness about the rational antibiotic use among pharmacy students and patient education by community pharmacists at the time antibiotics are dispensed to patients. Regulations are needed to restrict dispensing antibiotics without prescription among community pharmacists.

## Background

Antibiotic resistance is a major threat to public health worldwide. The World Health Organization (WHO) has described antibiotics resistance as a critical health problem that requires urgent actions to tackle [1]. Globally, antibiotic resistance is associated with mortality and deaths attributed to infections caused by antibiotic resistant pathogens is projected to reach 10 million deaths per year by the year 2050 [2]. Infections caused by multidrug resistant pathogens are associated with high mortality rate [3, 4]. The COVID-19 pandemic has caused an increase in the rate of multidrug resistant gram positive and gram-negative pathogens which escalates the burden of antibiotic resistance [5]. Antibiotic resistant pathogens are associated with both hospital and community acquired infections [3, 4, 6]. Antibiotic resistance is a consequence of the overuse, underuse, or misuse of antibiotics [1]. Inappropriate use of antibiotics has been reported among hospitalized patients and also in community setting [7, 8]. Self-medication with antibiotics and dispensing of antibiotics without prescription, especially in developing countries, contributes to the inappropriate use of antibiotics [9]. Dispensing antibiotics without prescription has been reported in previous studies [10, 11]. Evidence from a meta-analysis demonstrated that 62% of antibiotics are dispensed without a prescription in community pharmacy setting [12]. Antibiotics dispensed without prescription are used for the treatment of minor/common illnesses including upper respiratory tract infections and diarrhoea which do not need antibiotic therapy [12]. Antibiotics dispensed without prescription are associated with inappropriate use and sub-therapeutic dosing [13] leading to the emergence and spread of antibiotic resistance.

In Libya, self-medication with antibiotics is a common problem due to the lack of regulation restricting the over-the-counter sales of antibiotics [14]. In addition, dispensing antibiotics without prescription has been highlighted in a previous study and such practices led to inappropriate use of antibiotics [15]. Community pharmacies in Libya are identified as the main source of non-prescription antibiotics [15]. In addition, inappropriate practices among community pharmacists for the management of upper respiratory tract infections has been reported [16]. These practices contribute to the inappropriate use of antibiotics and the occurrence of antibiotic resistance. However, there is paucity of information describing the awareness, practices, and perceptions of community pharmacists towards antibiotic resistance and antibiotic stewardship. Antibiotic stewardship is an important strategy used in promoting the rational use of antibiotics and the involvement of community pharmacists is vital to the success of antibiotic stewardship programme in the community. The objective of this study is to evaluate the practices and perceptions of community pharmacists towards antibiotic use, antibiotic resistance, and strategies to improve appropriate use of antibiotics in Libya. Understanding these practices and perceptions is important to guide the design of interventions that will promote the involvement of community pharmacists in antibiotic stewardship programmes in Libya.

## Methods

### Study design and settings

This is a cross-sectional study conducted among community pharmacists practicing in Libya using an online, self-administered questionnaire. The questionnaire was distributed among the participants from December 2021 to February 2022. The study was conducted across all the cities in Libya.

### Inclusion and exclusion criteria

All practicing community pharmacists in community pharmacies across Libya were invited to participate in the study. Registered community pharmacists working in retail drug outlets who agreed to participate in the study were included. Non-community pharmacists such as medical practitioners and dentists were excluded to participate in this study.

### Study instrument

The study instrument (questionnaire) was developed after the review of previously published literature [10, 11, 17]. The questionnaire was developed in English language and validated by two lecturers who have expertise in questionnaire design. The items in the questionnaire were revised based on the comments and suggestions of the lecturers. The final version of questionnaire consisted of four sections including section A which collected the demographic information of the respondent (9 items), section B had 18 items that evaluated the practices and perceptions of community pharmacists towards antibiotic use and antibiotic resistance using a 5-point Likert scale (strongly agree, agree, neutral, disagree and strongly disagree). In section C, 11 items were used to assess the perception of community pharmacists towards antimicrobial stewardship (AMS). Section D had 9 items that focused on the perspectives of community pharmacists towards strategies to improve appropriate antibiotic use in community pharmacy. A 5-pointLikert scale was used in section D. The questionnaire was pre-tested among 10 community pharmacists and the internal consistency reliability alpha coefficients was 0.9.

### Data collection

Data collection was conducted using an online questionnaire. The hyperlink for the online questionnaire was shared with community pharmacists from December 2021 to February 2022. A snowball sampling method was using to recruit the participants. The community pharmacists initially recruited were asked to share the hyperlink for the online questionnaire with other community pharmacists in their network. The community pharmacists were invited to participate in the study through WhatsApp, Viber, and Messenger. Participation in the study was voluntary and the responses were unanimous. Reminders were sent to non-respondents after 2 weeks. The respondents were notified that the submission of responses was considered as consent to participate in the study. The study protocol was approved by ethical committee at Libyan International Medical University.

### Data analysis

Data were analysed using Statistical Package for Social Sciences software version 22. Categorical variables were presented as frequencies and percentages, while continuous variables were presented means and standard deviations. The responses in the practice and perception domains of the questionnaire were transformed into scores using 5 points for strongly agree to 1 point for strongly disagree. Negative items in the questionnaire were reverse coded. The responses in the Perspectives of community pharmacists towards strategies to improve appropriate antibiotic use in the community pharmacy were transformed into scores as follows: 5 points for very high priority and 1 point for not a priority. The total score in each domain was computed as the sum of the point for all the items in the domain. Mann Whitney (for two groups) and Kruskal Wallis (for three or more groups) tests were used to assess the differences in the scores based on the demographic characteristics of the participants. P value less than 0.05 was considered as statistical significance.

## Results

### Demographic characteristics

A total of 114 community pharmacists completed the questionnaire. Most of the respondents were female (54.4%), had less than 10 year working experience (78.1%), and had a Bachelor of Pharmacy as their highest qualification (81.6%). More than two-thirds (84.2%) worked in an independent community pharmacy, while 57.9% had another community pharmacy within 200 m radius away from their pharmacy. Table 1 summarizes the characteristics of the community pharmacists that participated in this study.

**Table 1:** Demographic characteristics of the community pharmacists

Variable	Frequency	Percentage
Location		
Benghazi	69	60.5
Derna	14	12.3
Tripoli	11	9.6
Tobruk	4	3.5
Alqubha	3	2.6
Sebha	2	1.8
Al- Majr	2	1.8
Others	7	6.1
Mean age (SD)	29.4 ± 4.6	
Gender		
Male	52	45.6
Female	62	54.4
Years of experience		
Less than 10 years	89	78.1
More than 10 years	24	21.1
Highest qualification		
Bachelor of Pharmacy	93	81.6
PharmD	10	8.8
Masters	6	5.3
Doctor of Philosophy (PhD)	1	0.9
Type of pharmacy		
Chain pharmacy	18	15.8
Independent pharmacy	96	84.2
Is there any community pharmacy within 200 m radius away from your pharmacy?		
Yes	66	57.9
No	25	21.9
I do not know	23	20.2
Number of pharmacists in community pharmacy		
Less than 10 pharmacists	105	92.1
More than 10 pharmacists	9	7.9
Do you have pharmacy assistants in your community pharmacy?		
Yes	49	43.0
No	58	50.9
I do not know	7	6.1

### Practices and perceptions of community pharmacists towards antibiotic use and antimicrobial resistance

#### Perception towards antimicrobial resistance

Most of the respondents (59.7%) strongly disagreed/disagreed that antibiotic resistance is a problem in the hospital setting and not a problem in the community. However, about one in five respondents (21%) strongly agreed/agreed with the same statement. More than two-thirds of the respondents strongly agreed/agreed that dispensing antibiotics without prescription from community pharmacy and the ease of obtaining antibiotics from community pharmacies contribute to the problem of antibiotic resistance. There was divergent response regarding the use of antibiotics for diarrhoea and sore throat. Less than 50% strongly disagreed/disagreed that antibiotics cure a patient with diarrhoea and sore throat faster than non-antibiotic treatment. More than two-thirds (78.1%) strongly agreed/agreed that community pharmacists have an important role to play to reduce antibiotic resistance. Overall, the participants have moderate perception towards antimicrobial resistance (median total perception score: 21.5; range: 16–28 out of a possible 35.0).

#### Practices towards antibiotic use and dispensing

More than 40% of the respondents indicated strongly agreed/agreed that they dispense broad spectrum antibiotics when they are in doubt which antibiotic is best for a patient and that they supply antibiotics when patients request them specifically, because if they do not supply them, patients will just go to another pharmacy. However, an overwhelming majority (86%) strongly agreed/agreed that it is important for pharmacists to only supply antibiotics when clinically needed, and not be driven by commercial pressures. More than two-thirds (71%) strongly agreed/agreed that it is important to supply a full course of antibiotics to a patient at the time, even when the patient says it is too expensive. However, about 47% indicated strongly agreed/agreed that if a patient cannot afford a full course of antibiotics all in one go, they will give them a smaller amount that they are able to afford at that time, even when a longer duration of treatment is required. Table 2 describes the perceptions and practices of community pharmacists towards antibiotic use and antimicrobial resistance.

**Table 2:** Perceptions and practices of community pharmacists towards antibiotic use and antimicrobial resistance

Variable	Frequency and percentage				
	Strongly disagree	Disagree	Neutral	Agree	Strongly agree
Perceptions towards antimicrobial resistance					
Antibiotic resistance is a problem in the hospital setting but not a problem in the community	40 (35.1)	28 (24.6)	22 (19.3)	13 (11.4)	11 (9.6)
Dispensing antibiotics without prescription from community pharmacies contributes to the problem of antibiotic resistance	5 (4.4)	6 (5.3)	14 (12.3)	18 (15.8)	71 (62.3)
The ease of availability of antibiotics from community pharmacies contributes to the problem of antibiotic resistance in Libya	2 (1.8)	6 (5.3)	21 (18.4)	27 (23.7)	58 (50.9)
Antibiotics cure a patient with diarrhoea more quickly than not having an antibiotic	29 (25.4)	25 (21.9)	33 (28.9)	17 (14.9)	10 (8.8)
Antibiotics give faster cure for a patient with a sore throat than non-antibiotic treatment	16 (14.0)	19 (16.7)	35 (30.7)	27 (23.7)	17 (14.9)
Antibiotic resistance resulting from the supply of antibiotics from community pharmacies is not a significant problem	47 (41.2)	14 (12.3)	19 (16.7)	18 (15.8)	16 (14.0)
Community pharmacists have an important role to play to reduce the problem of antibiotic resistance	3 (2.6)	13 (11.4)	9 (7.9)	13 (11.4)	76 (66.7)
Practices regarding antibiotic use					
If I am unsure whether or not a patient has a bacterial infection, I will supply antibiotics just in case it is	37 (32.5)	14 (12.3)	28 (24.6)	25 (21.9)	10 (8.8)
If I am in doubt which antibiotic is best for a patient, I will supply a broad spectrum one, just in case	18 (15.8)	13 (11.4)	31 (27.2)	39 (34.2)	13 (11.4)
I supply antibiotics when patients request them specifically, because if I do not supply them, they will just go to another pharmacy	31 (27.2)	18 (15.8)	14 (12.3)	24 (21.1)	27 (23.7)
It is important for pharmacists to only supply antibiotics when clinically needed, and not be driven by commercial pressures	3 (2.6)	9 (7.9)	4 (3.5)	31 (27.2)	67 (58.8)
When the pharmacy is busy, I am more likely to supply antibiotics if a customer asks specifically for an antibiotic, compared to when the pharmacy is quiet	29 (25.4)	14 (12.3)	26 (22.8)	26 (22.8)	19 (16.7)
It is important to supply a full course of antibiotics to a patient at the time, even when the patient says it is too expensive	2 (1.8)	10 (8.8)	21 (18.4)	34 (29.8)	47 (41.2)
I am happy to supply an antibiotic without further questioning if a patient requests one by name	28 (24.6)	26 (22.8)	14 (12.3)	34 (29.8)	12 (10.5)
I supply antibiotics only if I am certain that a patient has a bacterial infection	5 (4.4)	12 (10.5)	25 (21.9)	30 (26.3)	42 (36.8)
In cases where patients have no drug allergy history and no contraindication, I will supply a first line antibiotic as recommended in practice guidelines	9 (7.9)	14 (12.3)	33 (28.9)	36 (31.6)	22 (19.3)
If a patient cannot afford a full course of antibiotics all in one go, I will give them a smaller amount they are able to afford at that time, even when a longer duration of treatment is required	27 (23.7)	8 (7.0)	26 (22.8)	31 (27.2)	22 (19.3)
It is good practice for patients to keep a supply of antibiotics at home in case they need them later	49 (43.0)	21 (18.4)	19 (16.7)	11 (9.6)	14 (12.3)

### Community pharmacists’ involvement in antimicrobial stewardship programme

About two-thirds (66.7%) of the respondents had no previous involvement in antibiotic awareness campaign. The reasons for lack of involvement in previous campaigns included I have never heard about such campaign (48.7%) and I do not have enough time to participate (22.4%). An overwhelming majority (91.2%) indicated an interest in taken part in antibiotic awareness campaign to promote rational use of antibiotics (Table 3).

**Table 3:** Previous involvement in antibiotic awareness campaign among community pharmacists

Variable	Frequency	Percentage
Previous involvement in antibiotic awareness campaign		
Yes	38	33.3
No	76	66.7
Reasons for lack of previous involvement in antibiotic awareness campaign		
I have never heard about such campaigns	37	48.7
I do not have enough time to participate	17	22.4
I was not interested in participating in the campaign	1	1.3
There was no payment	–	–
Missing response	21	27.6
Are you interested in taken part in antibiotic awareness campaign to promote rational use of antibiotics?		
Yes	104	91.2
No	10	8.8

### Perceptions of community pharmacists towards antimicrobial stewardship

More than two-thirds of the respondents strongly agreed/agreed that AMS will reduce inappropriate use of antibiotics in the community and reduce antimicrobial resistance. Overall, the participants had a good perception towards antimicrobial stewardship (median total score: 20 [13–25] out of 25.0). More than two-thirds indicated strongly agreed/agreed that lack of knowledge about antibiotic resistance, lack of cooperation from healthcare professionals and lack of access to patients’ records hinder community pharmacists from participating in antimicrobial stewardship activities. Most of the respondents (> 80%) strongly agreed/agreed that raising awareness of rational antibiotic use including antimicrobial resistance, among pharmacy students and patient education by pharmacists in community pharmacies at the time are medicines are supplied to patients are strategies to improve appropriate use of antibiotics among patients in the community. Other strategies include providing regularly updated clinical practice guidelines to community pharmacies on the treatment of infectious diseases (78.5%, reclassification of all antibiotics as prescription-only medicine (74.5%) and raising awareness of rational antibiotic use including antimicrobial resistance, among the public through media, such as TV, radio and social media (77.2%). Table 4 shows the perception of community pharmacists towards antimicrobial stewardship.

**Table 4:** Perception of community pharmacists towards antimicrobial stewardship

Variable	Frequency and percentage				
	Strongly disagree	Disagree	Neutral	Agree	Strongly agree
Perception towards AMS					
AMS will reduce inappropriate use of antibiotics in the community	1 (0.9)	5 (4.4)	30 (26.3)	40 (35.1)	38 (33.3)
AMS will reduce the rate of antimicrobial resistance	1 (0.9)	5 (4.4)	28 (24.6)	43 (37.7)	37 (32.5)
AMS activities are important in the hospital setting but are not important in the community setting	40 (35.1)	32 (28.1)	23 (20.2)	13 (11.4)	6 (5.3)
Participation in AMS activities will boost public confidence in community pharmacy services	2 (1.8)	4 (3.5)	18 (15.8)	49 (43.0)	41 (36.0)
Community pharmacists’ participation in AMS programme will promote collaboration with physicians	2 (1.8)	10 (8.8)	18 (15.8)	37 (32.5)	47 (41.2)
Barriers to involvement in AMS					
Lack of knowledge about antibiotic resistance hinders community pharmacists from participating in AMS activities	2 (1.8)	11 (9.6)	25 (21.9)	42 (36.8)	34 (29.8)
Lack of cooperation from healthcare professionals prevents community pharmacists from participating in AMS activities	5 (4.4)	9 (7.9)	33 (28.9)	45 (39.5)	22 (19.3)
Lack of access to patient’s records limits community pharmacists’ involvement in AMS programme	6 (5.3)	8 (7.0)	25 (21.9)	40 (35.1)	35 (30.7)
Strategy to improve appropriate antibiotic use in the community pharmacy					
Patient education by pharmacists in community pharmacies at the time medicines are supplied to patients	1 (0.9)	7 (6.1)	13 (11.4)	46 (40.4)	47 (41.2)
Raising awareness of rational antibiotic use, including antimicrobial resistance, among the public through media, such as TV, radio, and social media	2 (1.8)	2 (1.8)	22 (19.3)	35 (30.7)	53 (46.5)
Educational programs of rational antibiotic use, including antimicrobial resistance, directed at the public	2 (1.8)	6 (5.3)	30 (26.3)	33 (28.9)	43 (37.7)
Raising awareness of rational antibiotic use, including antimicrobial resistance, among community pharmacists	2 (1.8)	6 (5.3)	20 (17.5)	37 (32.5)	49 (43.0)
Raising awareness of rational antibiotic use, including antimicrobial resistance, among pharmacy students	2 (1.8)	3 (2.6)	14 (12.3)	37 (32.5)	58 (50.9)
Providing regularly updated clinical practice guidelines to community pharmacies on the treatment of infectious diseases	3 (2.6)	9 (7.9)	12 (10.5)	47 (41.2)	43 (37.3)
Reclassification of all antibiotics as prescription-only	3 (2.6)	5 (4.4)	21 (18.4)	33 (28.9)	52 (45.6)
Enforcement of regulations which prohibit supply of antibiotics from non-pharmacies, and by non-pharmacists	2 (1.8)	9 (7.9)	13 (11.4)	38 (33.3)	52 (45.6)
Motivation of community pharmacists to be involved in antibiotic use campaigns through providing monetary incentives	1 (0.9)	7 (6.1)	21 (18.4)	48 (42.1)	37 (32.5)

### Differences in total score for perception towards antimicrobial resistance and antimicrobial stewardship and total practice score for antibiotic use among community pharmacists

Female participants (37.0 [23–49] vs. 35.5 [25–48]; *p* = 0.005), those who worked in chain pharmacy (37.0 [23–49] vs. 33.0 [27–47]; *p* = 0.019), those who had no pharmacy assistant (38.5 [27–47) vs. 34.0 [23–49); p = 0.025) and those who had no/I do not know previous involvement in antibiotic awareness campaign (37.5 [23–49] vs. 32.0 [25–48]; *p* < 0.001) had better score regarding antibiotic practices compared to their other counterparts. Female participants (22.0 [17–29] vs. 21.5 [15–28]) and those who had no/I do not know previous involvement in antibiotic awareness campaign (22.0 [15–29] vs. 21.0 [16–28]; *p* = 0.006) had significantly better perception towards antimicrobial resistance than males and those who had previous involvement in antibiotic awareness campaign. Those who had undergraduate qualification (20.0 [13–25] vs. 19.0 [13–24]; *p* = 0.013) and those who had no/I do not know previous involvement in antibiotic awareness campaign (20.0 [13–25] vs. 19.0 [13–25]; *p* = 0.032) had better perception towards antimicrobial stewardship (Table 5).

**Table 5:** Differences in the total median score for perception towards antimicrobial resistance and antimicrobial stewardship and total score for practices towards antibiotic use among community pharmacists

Variable	Median total perception towards AMR (IQR)	P value	Median total practices towards antibiotic use (IQR)	P value	Median total perception towards AMS (IQR)	P value	Median total perception towards AMS strategies (IQR)	P value
Gender								
Male	21.5 (15–28)	**0.011**	35.5 (25–48)	**0.005**	20.0 (13–25)	0.293	37.0 (25–45)	0.963
Female	22.0 (17–29)		37.0 (23–49)		20.0 (13–25)		36.0 (18–45)	
Years of experience								
Less than 10 years	22.0 (15–29)	0.209	36.5 (23–49)	0.418	20.0 (13–25)	0.294	36.0 (18–45)	0.380
More than 10 years	24.0 (18–28)		37.0 (31–38)		21.0 (13–25)		39.0 (31–44)	
Highest qualification								
Undergraduate degree	22.0 (15–28)	0.331	37.0 (23–49)	0.260	20.0 (13–25)	**0.013**	37.0 (18–45)	0.903
Postgraduate degree	20.0 (18–29)		36.5 (25–45)		19.0 (13–24)		36.5 (25–45)	
Type of pharmacy								
Chain pharmacy	21.5 (15–25)	0.150	**37.0 (23–49)**	**0.019**	20.0 (13–25)	0.363	36.0 (23–45)	0.242
Independent pharmacy	22.0 (15–29)		**33.0 (27–47)**		20 (13–25)		37.0 (18–45)	
Is there any community pharmacy within 200 m radius away from your pharmacy?								
Yes	22.0 (15–29)	0.407	37.0 (23–49)	0.789	20.0 (13–25)	0.137	36.0 (18–45)	0.508
No/I do not know	22.0 (16–28)		36.5 (27–48)		19.0 (13–25)		38.0 (20–45)	
Do you have pharmacy assistants in your community pharmacy?								
Yes	22.0 (15–28)	0.341	**34.0 (23–49)**	**0.025**	20.0 (13–25)	0.664	37.0 (27–45)	0.761
No/I do not know	22.0 (18–29)		**38.5 (27–47)**		20.0 (13–25)		36.0 (18–45)	
Previous involvement in antibiotic awareness campaign								
Yes	**21.0 (16–28)**	**0.006**	**32.0 (25–48)**	** < 0.001**	**19.0 (13–25)**	**0.032**	35.0 (20–45)	0.090
No	**22.0 (15–29)**		**37.5 (23–49)**		**20.0 (13–25)**		36.5 (18–45)	
Are you interested in taken part in antibiotic awareness campaign to promote rational use of antibiotics?								
Yes	22.0 (15–29)	0.471	36.0 (25–49)	0.845	20.0 (13–25)	0.356	36.0 (18–25)	0.907
No	23.0 (20–24)		37.0 (23–47)		21.0 (14–25)		37.0 (33–44)	

Bold font denotes statistical significance

## Discussion

This study evaluated the knowledge, attitude, and perception of Libyan community pharmacists towards antibiotic use, antibiotic resistance and antibiotic stewardship. The results showed that most community pharmacists, who are the only expert who dispensed medication along with hospital pharmacists, were aware that their activities including dispensing antibiotics without prescriptions and supplying shorter course of antibiotics to customer who cannot afford a full course of treatment contributes to the emergence and spread of antibiotic resistance. In addition, community pharmacists were aware that they have an important role to play in the fight against antibiotic resistance. These findings are consistent with previous studies conducted in Nigeria and Saudi Arabia [10, 18]. A previous study demonstrated that knowledge of the negative implications of dispensing antibiotics without prescriptions does not deter community pharmacists from such malpractices [10]. This highlights the need for the enforcement of regulations that prohibits dispensing antibiotics without prescriptions in community pharmacy setting. The results revealed that there were more community pharmacists who perceive the need for antibiotics to cure sore throat (more than one-thirds 38.6%) than there were for diarrhoea (less than one-thirds 23.7%). This implies that community pharmacists are more likely to dispense antibiotics for the treatment of upper respiratory tract infections (URTI) than diarrhoea, similar to the result of previous studies [10, 18]. URTI is one of the most common infections that does not require antibiotic treatment for which community pharmacists commonly dispense antibiotics without treatment [12]. Overall, community pharmacists were found to have a moderate perception towards antimicrobial resistance, and this could influence their practices towards antibiotic use and dispensing. These findings indicate that community pharmacists need more training to improve their knowledge and perception regarding the treatment of infections that do not require antibiotics. In addition, antibiotic resistance and antibiotic use training should be extended to undergraduate and postgraduate pharmacy students to prepare them for future practice as recommended in previous studies [19–21].

Majority of the participants (86%) strongly agreed/agreed that community pharmacists should only supply antibiotics when there is a clinical indication and that they should not be driven by commercial pressure. However, less than half (44.8%) strongly disagreed/disagreed to supply antibiotics if there was uncertainty whether or not a patient has a bacterial infection. About one-thirds strongly agreed/agreed to supply antibiotics in situations, where it is unclear if a patient has a bacterial infection. This inappropriate antibiotic dispensing practice is driven by a number of factors including patient pressure, for example, patients requesting for a specific antibiotic by name; fear of losing customers to another pharmacy upon refusal to supply antibiotics; and busy pharmacy working hours. These findings are in consonance with the results of previous studies [22, 23]. This implies that both patient-related and community pharmacists-related factors contribute to the inappropriate use of antibiotics in the community. Therefore, antimicrobial stewardship interventions to improve appropriate use of antibiotics in the community should be targeted at both the public and the community pharmacists.

Dispensing antibiotics without prescription is associated with inappropriate use of antibiotics including sub-therapeutic dosing [13]. The current study showed that almost 50% (46.5%) strongly agreed/agreed to supply shorter course of antibiotic therapy when a patient cannot afford a full course of antibiotics all in one go. This inappropriate among community pharmacists cannot be attributed to lack of awareness, because more than two-thirds of them strongly agreed/agreed that it is important to supply a full course of antibiotics to a patient at the time, even when the patient says it is too expensive. This malpractice demonstrates that community pharmacists prioritised financial gains over the risk of antibiotic resistance. Therefore, public awareness about rational use of antibiotics including the use of full course of antibiotics for infections are recommended to promoted patients’ participation in the fight against antibiotic resistance and reduce customer pressure on community pharmacists.

Evidence has shown that antibiotic stewardship interventions are effective in promoting appropriate use of antibiotics and improving clinical outcomes in hospital setting [24]. In addition, pharmacists are important members of a multidisciplinary antibiotic stewardship team [17]. In the current study, the results demonstrated that only one-thirds of the community pharmacists had previous involvement in antibiotic awareness campaigns. A previous study revealed limited pharmacists’ involvement in antimicrobial stewardship program in hospital setting [25]. The main reasons for non-involvement in antibiotic awareness campaigns were lack of awareness and lack of enough time to participate. It is interesting to note that an overwhelming majority of community pharmacists indicated interest in participating in antibiotic awareness campaign to promote rational use of antibiotics. Currently, there is no mandatory courses for continuing professional development points in Libya. However, awareness lectures are organized for community pharmacist to improve their knowledge and skills, but this is not obligatory. Therefore, strategies to promote community pharmacists' involvement in antibiotic awareness campaign should be targeted at adequate dissemination information about such campaigns through channels that would reach as many community pharmacists as possible including community pharmacists' associations or societies.

Community pharmacists have an important role to play in promoting rational use of antibiotics among the public due to their easy access to the public. The most common strategies supported by community pharmacists were raising awareness of rational antibiotic use among pharmacy students, patient education by community pharmacists at the time antibiotics are dispensed to patients and raising awareness of rational antibiotic use and antimicrobial resistance among the public through mass media. These strategies are like the first objective of the global action plan for antimicrobial resistance which recommends raising awareness and understanding of antimicrobial resistance through effective communication, education, and training [26]. In addition, evidence has shown that patient education at the time of dispensing antibiotics significantly improved treatment adherence compared to usual care [27]. Another study revealed that a tailored educational intervention significantly improved public knowledge about antibiotic use [28]. Other strategies include reclassification of all antibiotics as prescription-only medicines and enforcement of regulation against dispensing of antibiotics by non-pharmacists and from non-pharmacies. These strategies will reduce dispensing of antibiotics without prescription resulting in a decline in self-medication with antibiotics and inappropriate use of antibiotics associated with self-medication. In Saudi Arabia, the enforcement of a regulation prohibiting the dispensing antibiotics without prescription significantly reduced such malpractice among community pharmacist [29]. Therefore, the enforcement of similar regulations is recommended in Libya to promote rational use of antibiotics among the public.

This study has some limitations; therefore, the results should be interpreted with caution. First, the study population is relatively small, and this may affect the generalizability of the results. However, an exhaustive recruitment technique was used including sharing the hyperlink to the survey through the social media platforms for community pharmacists in Libya. In addition, the study included respondents from more than 10 states in Libya which increases the generalizability of the findings. Second, the responses were self-reported, and these are susceptible to social desirability bias. Despite the limitations, the study provides an insight into the perceptions and practices of community pharmacists in Libya towards antibiotic use, antibiotic resistance, and antibiotic stewardship. In addition, the study provides some information on the strategies to improve rational use of antibiotics among the public.

## Conclusion

Community pharmacists in Libya recognised that they have an important role to play in promoting rational use of antibiotics. Community pharmacists had a moderate perception towards antimicrobial resistance and a good perception towards antimicrobial stewardship. However, inappropriate antibiotic practices were common including dispensing antibiotics upon request by patients and supplying shorter course of antibiotics for patients who cannot afford a full course of treatment are common. The most common strategies to improve rational use of antibiotics in community pharmacy were raising awareness about the rational antibiotic use among pharmacy students, patient education by community pharmacists at the time antibiotics are dispensed to patients and raising awareness about the rational antibiotic use and antimicrobial resistance among the public through mass media.

## Data Availability

All data generated or analysed during this study are included in this published article.

## Abbreviations

AMR: Antimicrobial resistance
AMS: Antimicrobial stewardship
IQR: Interquartile range
SD: Standard deviation
URTI: Upper respiratory tract infection
WHO: World Health Organisation

## References

[1] World Health Organization. Antimicrobial resistance: global report on surveillance. World Health Organization; 2014. Published April 2014. https://www.who.int/antimicrobial-resistance/publications/surveillancereport/en/. Accessed 4 Dec 2021.

[2] O’Neill J. Review on antimicrobial resistance antimicrobial resistance: tackling a crisis for the health and wealth of nations. London: Review on Antimicrobial Resistance; 2014. https://amr-review.org/sites/default/files/AMR%20Review%20Paper%20%20Tackling%20a%20crisis%20for%20the%20health%20and%20wealth%20of%20nations_1.pdf. Accessed 10 Mar 2022.

[3] Abubakar U, Tangiisuran B, Elnaem MH, Sulaiman SA, Khan FU (2022). Mortality and its predictors among hospitalized patients with infections due to extended spectrum beta-lactamase (ESBL) Enterobacteriaceae in Malaysia: a retrospective observational study. Futur J Pharm Sci.

[4] Abubakar U, Zulkarnain AI, Rodríguez-Baño J, Kamarudin N, Elrggal ME, Elnaem MH, Harun SN (2022). Treatments and predictors of mortality for carbapenem-resistant gram-negative bacilli infections in Malaysia: a retrospective cohort study. Trop Med Infect Dis.

[5] Abubakar U, Al-Anazi M, Rodríguez-Baño J (2023). Impact of COVID-19 pandemic on multidrug resistant gram positive and gram negative pathogens: a systematic review. J Infect Public Health.

[6] Abubakar U, Amir O, Rodríguez-Baño J (2022). Healthcare-associated infections in Africa: a systematic review and meta-analysis of point prevalence studies. J Pharm Policy Pract.

[7] Abubakar U, Syed Sulaiman SA, Adesiyun AG (2018). Utilization of surgical antibiotic prophylaxis for obstetrics and gynaecology surgeries in Northern Nigeria. Int J Clin Pharm.

[8] Abubakar U (2020). Antibiotic use among hospitalized patients in northern Nigeria: a multicenter point-prevalence survey. BMC Infect Dis.

[9] Roca I, Akova M, Baquero F, Carlet J, Cavaleri M, Coenen S, Cohen J, Findlay D, Gyssens I, Heure OE, Kahlmeter G (2015). The global threat of antimicrobial resistance: science for intervention. New Microbes and New Infections.

[10] Abubakar U, Tangiisuran B (2020). Knowledge and practices of community pharmacists towards non-prescription dispensing of antibiotics in Northern Nigeria. Int J Clin Pharm.

[11] Abubakar U (2020). Practices and perceptions of Nigerian community pharmacists toward antimicrobial stewardship program. Int J Pharm Pharm Sci.

[12] Auta A, Hadi MA, Oga E, Adewuyi EO, Abdu-Aguye SN, Adeloye D, Strickland-Hodge B, Morgan DJ (2019). Global access to antibiotics without prescription in community pharmacies: a systematic review and meta-analysis. J Infect.

[13] Esimone CO, Nworu CS, Udeogaranya OP (2007). Utilization of antimicrobial agents with and without prescription by out-patients in selected pharmacies in South-eastern Nigeria. Pharm World Sci.

[14] Ghaieth MF, Elhag SR, Hussien ME, Konozy EH (2015). Antibiotics self-medication among medical and nonmedical students at two prominent Universities in Benghazi City, Libya. J Pharm Bioallied Sci.

[15] Atia A (2018). Monitoring the level of antibiotic purchase without a prescription among Libyan young adults. Indian J Pharm Pract.

[16] Atia AE, Abired AN (2017). Antibiotic prescribing for upper respiratory tract infections by Libyan community pharmacists and medical practitioners: an observational study. Libyan J Med Sci.

[17] Donsamak S. Exploring factors that influence the supply and use of antibiotics from community pharmacies in Thailand (Doctoral dissertation, Cardiff University).

[18] Hadi MA, Karami NA, Al-Muwalid AS, Al-Otabi A, Al-Subahi E, Bamomen A, Mohamed MM, Elrggal ME (2016). Community pharmacists’ knowledge, attitude, and practices towards dispensing antibiotics without prescription (DAwP): a cross-sectional survey in Makkah Province, Saudi Arabia. Int J Infect Dis.

[19] Abubakar U, Muhammad HT, Sulaiman SA, Ramatillah DL, Amir O (2020). Knowledge and self-confidence of antibiotic resistance, appropriate antibiotic therapy, and antibiotic stewardship among pharmacy undergraduate students in three Asian countries. Curr Pharm Teach Learn.

[20] Abubakar U, Sha’aban A, Mohammed M, Muhammad HT, Sulaiman SA, Amir O (2021). Knowledge and self-reported confidence in antimicrobial stewardship programme among final year pharmacy undergraduate students in Malaysia and Nigeria. Pharm Educ.

[21] Khan FU, Khan A, Shah S, Hayat K, Usman A, Khan FU, Khan Z, Karataş Y, Ahmad T, Chang J, Malik UR (2021). Exploring undergraduate pharmacy students perspectives towards antibiotics use, antibiotic resistance, and antibiotic stewardship programs along with the pharmacy teachers’ perspectives: a mixed-methods study from Pakistan. Front Pharmacol.

[22] Zawahir S, Lekamwasam S, Aslani P (2019). A cross-sectional national survey of community pharmacy staff: Knowledge and antibiotic provision. PLoS ONE.

[23] Zapata-Cachafeiro M, González-González C, Váquez-Lago JM, López-Vázquez P, López-Durán A, Smyth E, Figueiras A (2014). Determinants of antibiotic dispensing without a medical prescription: a cross-sectional study in the north of Spain. J Antimicrob Chemother.

[24] Abubakar U, Syed Sulaiman SA, Adesiyun AG (2019). Impact of pharmacist-led antibiotic stewardship interventions on compliance with surgical antibiotic prophylaxis in obstetric and gynecologic surgeries in Nigeria. PLoS ONE.

[25] Abubakar U, Tangiisuran B (2020). Nationwide survey of pharmacists’ involvement in antimicrobial stewardship programs in Nigerian tertiary hospitals. J Glob Antimicrob Resist.

[26] World Health Organization. Global action plan on antimicrobial resistance. World Health Organization, 2015. https://www.who.int/publications/i/item/9789241509763. Last accessed 17 Mar 2023.

[27] Munoz EB, Dorado MF, Guerrero JE, Martínez FM (2014). The effect of an educational intervention to improve patient antibiotic adherence during dispensing in a community pharmacy. Aten Primaria.

[28] Shehadeh MB, Suaifan GA, Hammad EA (2016). Active educational intervention as a tool to improve safe and appropriate use of antibiotics. Saudi Pharm J.

[29] Alrasheedy AA, Alsalloum MA, Almuqbil FA, Almuzaini MA, Aba Alkhayl BS, Albishri AS, Alharbi FF, Alharbi SR, Alodhayb AK, Alfadl AA, Godman B (2020). The impact of law enforcement on dispensing antibiotics without prescription: a multi-methods study from Saudi Arabia. Expert Rev Anti Infect Ther.

